# A co-production study of an art-based media campaign intervention to encourage early breast cancer screening uptake among Black women in England

**DOI:** 10.3389/fpubh.2026.1875785

**Published:** 2026-09-03

**Authors:** Tarela Juliet Ike, Dung Ezekiel Jidong, Mieyebi Lawrence Ike, Evangelyn Ebi Ayobi, Peremi Richmond Ike, Oluwakorede Florence Adebowale, Gabriella Rooks, Nusrat Husain

**Affiliations:** 1 Division of Psychology and Mental Health, University of Manchester, Manchester, United Kingdom; 2 School of Social Sciences, Humanities, and Law, Teesside University, Middlesbrough, United Kingdom; 3 Division of Psychology and Mental Health, University of Manchester, Manchester, United Kingdom; 4 Western Governors University, Millcreek, UT, United States; 5 Manchester Global Foundation, Manchester, United Kingdom; 6 Beryl Foundation, London, United Kingdom; 7 Moonrise 24 Hrs Recruitment, Stockton-on-Tees, United Kingdom; 8 Division of Psychology and Mental Health, University of Manchester, Manchester, United Kingdom; 9 Mersey Care NHS Foundation Trust, Prescot, United Kingdom

**Keywords:** Black women, breast cancer, early screening uptake, England, United Kingdom

## Abstract

**Background:**

Breast cancer is one of the most common cancers that affects women globally. Of central concern are Black women in the United Kingdom who are disproportionately affected, present late and suffer higher mortality due to poor early screening uptake. Informed by an interpretative phenomenological analytical lens, this study makes an original and significant contribution to address the gap by working with 15 Black women from the north of England to co-produce an art-based media campaign intervention designed to encourage early breast cancer screening uptake.

**Method:**

*N* = 10 participants took part in a focus group discussion, while *N* = 5 took part in individual interviews. Data were analyzed using interpretative phenomenological analysis.

**Findings:**

The following main themes emerged: (i) Uncertainty underpinned by poor awareness, (ii) Fear of the unknown, (iii) From fear to action: Empowerment-focused breast screening communication, (iv) Animated content, (v) Embedding awareness in women’s everyday digital spaces, and (vi) Empowerment through coproduction, inclusive voice and agency.

**Conclusion:**

The study has important policy implications, including the need for co-designed interventions to foster uptake, and it also recommends testing the co-produced intervention to determine its feasibility, effectiveness, and cost-effectiveness in encouraging early screening uptake among this marginalized population.

## Introduction

Breast cancer is a significant public health concern which mostly affects women. Data from the World Health Organization (1) suggest that globally, an estimated 2.3 million women are diagnosed with breast cancer, with 670,000 deaths reported. In the United Kingdom, breast cancer is the most common form of cancer, resulting in an estimated 11,200 deaths yearly and equating to 31 deaths daily (2). Of central concern are black women in the United Kingdom who are more severely affected. Existing data suggest that breast carcinoma *in situ* incident rates are lower in females from Asian backgrounds, including people of mixed or multiple ethnicities, but higher in the Black ethnic group, compared to females of White ethnicity in England (2). Black women in the United Kingdom are also often diagnosed younger, with the median age of diagnosis at 58 compared to 62 years for White women and are also up to 71% more likely to be diagnosed with the disease at a late stage—thereby facing higher mortality risk and more aggressive subtypes (3). Yet a gap remains in intervention to address this problem for black women using a coproduced media campaign intervention to encourage early screening uptake and to reduce the risk of late presentation and mortality.

A growing body of literature has often focused on the factors that underpin the pattern of late diagnosis (4, 5). A major contributor is reduced participation in breast cancer screening programs, partly attributed to poor awareness (6). Research consistently shows that Black women in the United Kingdom are less likely to attend mammography appointments, despite screening being proven to improve survival by enabling early detection (7). Lower screening uptake is also argued to be characterized by lower awareness of breast cancer symptoms and risk factors across ethnic minority communities (8). Data from Cancer Research UK (9) indicate that women from minority backgrounds have significantly lower knowledge of cancer symptoms and are less confident discussing concerns with health professionals, reducing the likelihood of early help-seeking. Cultural factors—such as stigma (10), misconceptions (8), and silence surrounding cancer (5)—also contribute to fear, avoidance, and delayed medical engagement. Some communities perceive cancer as a death sentence or, in some cases, believe it might be contagious, fostering reluctance to disclose symptoms or seek screenings (11). Communication barriers and a lack of culturally attuned healthcare interactions further exacerbate these problems (8, 12), with language difficulties and limited trust in providers restricting engagement with cancer services (13).

Socioeconomic inequality adds another dimension to late diagnosis. Black women, as part of ethnic minorities, are more likely to reside in deprived areas, where late-stage presentation is more common (14). Prevent Breast Cancer reports that breast cancer mortality rates are 17% higher for women residing in the most deprived areas, a pattern in which Black women are overrepresented (15). Structural inequalities (16), mistrust in public services (17), including healthcare, and differences in beliefs about breast cancer collectively shape diagnostic outcomes for Black women. In addition, systemic issues within healthcare—including insufficiently tailored awareness campaigns (8) and inadequate provider understanding of Black patients’ needs—further reinforce these inequities. This concern is further amplified by gaps in research on the co-design of targeted interventions to address poor screening uptake.

The present study addresses a gap in the preceding literature by adopting Interpretative Phenomenological Analysis (IPA), a rigorous and highly relevant methodological lens for co-producing an art-based media intervention to encourage early screening uptake among Black women in the United Kingdom. Because IPA prioritizes lived experience and personal meaning-making (18), it offers a unique avenue for uncovering how Black women interpret their identities, cultural contexts, and interactions with the healthcare system. This is especially important given that many barriers to screening—including mistrust, stigma, cultural silence, misconceptions, and feelings of invisibility within public health communication—appear rooted in structural constraints as well as in personal and collective narratives that shape health behaviors. By enabling deep engagement with the relational, symbolic and emotional dimensions of women’s screening decisions, IPA could help reveal precisely those insights that traditional survey-based or biomedical approaches often overlook.

Furthermore, when embedded within a co-production process, the phenomenological insights of IPA function as creative foundations for an art-based intervention, allowing Black women to collaboratively shape messages, imagery, and narratives that reflect their cultural identities, esthetic sensibilities, concerns, and hopes. This not only enhances the campaign’s authenticity and emotional resonance but also ensures it directly responds to the specific lived experiences that contribute to delayed screening. In this way, IPA provides both an original and significant contribution to intervention design by empowering Black women as equal partners in knowledge creation, ultimately increasing the potential for meaningful change in screening behaviors. Against the preceding backdrop, the study addressed the following research question:

- How can Black women who are partially or not up to date with breast cancer screening inform the development and co-production of an art-based media intervention that effectively promotes early screening uptake in the United Kingdom?

To address the research question, the next section details the methodology adopted. This is followed by the findings and a discussion linked to the literature.

## Methodology

This study adopted a case study design informed by a qualitative research approach, underpinned by an interpretivist epistemology and a constructionist ontology, allowing for an in-depth exploration of how Black women make meaning of low breast cancer screening uptake within their sociocultural contexts and co-produce interventions to address the issue. From an interpretivist stance, knowledge is understood as emerging from participants’ subjective experiences and the researcher’s interpretive engagement with those experiences (19), which aligns well with the use of Interpretative Phenomenological Analysis (18). The constructionist ontological position further recognizes that participants’ realities are socially produced—shaped by culture, community narratives, health-system interactions, and lived histories (20)—thereby enabling the study to capture how black women construct personal and collective understandings of fear, awareness, and representation in screening. By combining these methodological commitments within a case study framework, the research generated rich, situated insights into a specific community context while acknowledging the multiple social forces that shape meaning-making.

### Ethics statement

Ethical approval for this study was sought for and granted by the Teesside University School of Social Sciences, Humanities and Law Research Ethics Sub-Committee. The study adhered to established guidelines for conducting qualitative research with human participants. In keeping with the study’s interpretivist and constructionist foundations, ethical practice ensured procedural compliance and also included ongoing reflexivity, respect for participant autonomy, and sensitivity to the cultural context of Black women’s experiences of breast-cancer screening. Informed consent was obtained prior to participation, with clear explanations of the study aims, the voluntary nature of involvement, the right to withdraw, and how confidentiality and data protection would be maintained. Given the potentially sensitive nature of discussing fear, stigma, and healthcare experiences, the research design prioritized emotional safety by providing participants with choice, control, and supportive communication throughout the coproduction process. This approach ensured an inclusive ethos that valued participants as co-creators of knowledge. The study was also conducted in accordance with the Declaration of Helsinki, and participants were informed about how their data would be used, including for publication and presentation.

### Participant demographics and sampling

A purposive sampling strategy was employed to recruit participants who met the inclusion criteria in line with the study aims. The inclusion criteria are that a participant is eligible if they are not or partially up to date with breast cancer screening, able to give informed consent and are aged 18 years and above. The reason for the age range is that while breast cancer mostly affects women over 50 years, studies have also shown that younger women can also be affected. For example, data from Breast Cancer Now UK (21) indicates that around 5,000 women aged 45 or younger are diagnosed with breast cancer in the United Kingdom each year. In essence, our study aims to capture the entire age range, in line with its goal of aiding the co-production of an intervention to encourage early screening uptake. Prospective participants were excluded if they did not meet the inclusion criteria. Recruitment drew on established relationships with third-sector organizations, including non-governmental and faith-based groups such as churches and mosques, which provided access to women who reflect the communities under study. To enhance reach and ensure inclusion of individuals with similar lived experiences, participants also suggested others who might be willing to take part, enabling a focused yet diverse sample while maintaining the intentionality central to purposive sampling.

In total, 15 females with lived experiences of not being up to date or partially up to date or partially up to date with breast cancer screening were recruited for the study. Of the 15, *n* = 3 indicated a secondary level of education, while the other 12 indicated tertiary level education. Regarding employment status, 11 were employed, while the other 4 were unemployed. The participants were recruited from England, including Middlesbrough, Hartlepool, Redcar and Cleveland, Manchester, and the Tees Valley. The participants’ ages ranged from 18 to 65 years.

### Data collection

To support and strengthen the data-collection process, the study first established a steering committee composed of individuals with relevant lived experience, who played a central role in shaping the initial focus group questions. In addition, an early round of stakeholder engagement was conducted with black women who had lived experiences of not engaging in breast cancer screening exercises; these stakeholders underscored the importance of developing an art-based media campaign intervention and advised on the types of questions needed to ensure the campaign would be coherent, culturally appropriate, and responsive to the needs of the affected population. Their collective insights directly informed the development of the focus group guide and interview schedule, which were subsequently submitted for ethical review and approved by the first author’s university.

Examples of questions contained in the focus group guide include: What are your experiences of breast cancer screening uptake? What do you think causes poor early breast cancer screening uptake for black women in the United Kingdom? What are some of the effects of poor breast cancer screening uptake? Based on your responses to the previous questions, what factors should we consider when designing the art-based media campaign intervention? What do you suggest the content (e.g., introduction, main body and conclusion) of the art-based media campaign intervention should include? What sort of artwork would you suggest? What length do you propose the art-based media campaign intervention could be, and why? What social media platform would you encourage to disseminate the art-based media campaign intervention and why? What do you think are the barriers that could potentially prevent the success of the art-based media campaign intervention? What do you think are the factors that need to be considered to ensure the successful implementation of the proposed intervention? Who are the key people (e.g., stakeholders, organizations) that need to be involved to make the outcome of the intervention a success? Are there any thoughts you would like to add that may not have been covered in this focus group?

In total, one focus group involving 10 participants was conducted. The session was facilitated by a trained black female with relevant lived experience, who had received additional instruction from the research team in focus-group interviewing techniques. To ensure methodological rigor and provide further support, the facilitator was accompanied by an experienced black senior researcher of African descent—also with lived experience of working with the affected population—as well as other senior members of the project team, including a psychologist. This combination of lived expertise and professional research experience strengthened the quality, sensitivity, and depth of the data collected. In addition to the focus group, five individual interviews were conducted with participants via Microsoft Teams.

### Data analysis

The recorded focus group and individual interviews were transcribed verbatim into a Word document. Members of the steering group were trained in interpretative phenomenological analysis by a senior member of the research team with a doctoral (PhD) qualification. The data were analyzed using Interpretative Phenomenological Analysis (IPA) (18), which aligned with the study’s interpretivist epistemology and constructionist ontology by enabling a deep exploration of how Black women made sense of breast cancer screening through their lived experiences and co-producing the solution to poor early screening uptake. The analysis began with repeated, immersive readings of each transcript to ensure close engagement with participants’ words, tone, metaphors, and experiential claims. Following IPA’s idiographic commitment, each case was analyzed individually before any cross-case patterns were explored. Detailed exploratory comments were developed at three levels: descriptive (attending to what participants said about fear, awareness, and representation), linguistic (examining language choices such as hesitations, emphases, and culturally meaningful expressions), and conceptual (drawing out interpretative insights regarding meaning-making, identity, and emotional embeddedness). From these layered annotations, emergent themes were generated that captured the essence of each participant’s account. Only after each individual extract had been analyzed independently were the themes compared across cases to develop superordinate themes that reflected shared experiences while preserving the nuance and diversity of individual meaning, particularly around cultural identity, digital media use, and the emotional framing of breast cancer.

To enhance analytic rigor, triangulation, member checking and intercoder reliability were incorporated in line with IPA’s interpretative nature. Two trained members of the steering group independently reviewed a subset of transcripts, exploratory notes, and emerging themes. The process focused on coherence, interpretative convergence, and grounding in the data. Any areas of divergence between analysts became opportunities for reflexive dialog, which deepened interpretative insight and supported a transparent justification of analytic decisions. This iterative and collaborative checking safeguarded against individual researcher bias while maintaining IPA’s commitment to multiple layers of meaning. It is also important to note that given the qualitative nature of the study, there were no missing data, and incomplete sentences were followed during the focus group to ensure accuracy. To enhance rigor and reflectivity, reflexive memos documenting interpretative decisions, theme development, and analytic shifts were reviewed by the team and further strengthened the dependability and trustworthiness of the analysis, ensuring that the final thematic structure authentically represented how Black women in the coproduction process constructed, negotiated, and expressed their understandings of an art-based media intervention to encourage breast-cancer screening uptake. Below is an example of a table summarizing superordinate themes and representative quotes (see Table 1).

**Table 1:** Summary themes illustrating examples of representative quotes and superordinate themes.

Transcripts	Emergent themes	Clustered superordinate themes
“For me I think it’s a mix of lack of awareness, fear, cultural beliefs and how the healthcare system interacts with black women” (Kaisha)	- Poor awareness	Fear of the unknown
“So, for me, I have not done one and I really think […] is due to fear. So, and fear and lack of information really. So back home in Nigeria, I am not sure. There wasn’t quite a lot of information on you going for breast cancer screening, but when I got here. and I got a letter to go for a cervical cancer screening I was confused and I never got any regarding breast cancer. (Omolola)”	- Uncertainty	
	- Fear	
“I believe it’s; I feel personally it was very helpful because lack of awareness is something that came up very frequently and not knowing what to do, even if you have something, even if you have a concern about your breast or anything. So, this was really helpful and getting the input and knowing what would help to reach out to people to get aware of what is going on is really good.” (Brianna)	-Sense of empowerment	Empowerment and meaningful message underpinned by coproduced experience
“Overall, being part of this coproduction process makes me feel part of the solution to poor early breast cancer screening for black women. It gives me a sense of ownership to contribute to the solution, especially given my lived experience of never having gone for breast cancer screening. I am truly happy to be part of this inclusive process that takes a bottom-up approach and gives a voice to black women like me, who are often not heard.” (Teyana)	- Ownership	
	- Relevance	

## Findings

Based on the analyzed data underpinned by IPA the following main superordinate themes were reported: (i) Uncertainty underpinned by poor awareness, (ii) Fear of the unknown, (iii) From fear to action: Empowerment-focused breast screening communication, (iv) Animated content, (v) length, (vi) Embedding Awareness in Women’s everyday digital spaces, and (vii) Empowerment through coproduction, inclusive voice and agency.

### Uncertainty underpinned by poor awareness

Ensuring early breast cancer screening uptake requires effective awareness creation. A notable pattern in the participants’ lived experiences was perceived uncertainty, characterized by poor awareness, thereby precluding them from engaging in early screening. Such poor awareness was perceived from a micro and macro systemic perspective. Talking about this, one participant said:

The reason I have not had any […], I will say, I have not gotten like an information about it. Like, okay, is there a campaign? Is there something going on that is saying, oh, ladies, you can go for breast cancer at so so-so place? Is it at our GPs we are supposed to do it? I do not have that information. So, that would be a reason why I have not done any (Asante).

The participant constructs breast cancer screening not as a personal neglect or unwillingness, but as a consequence of perceived systemic communication failures. Her repeated references to not having *“gotten…information”* and her series of unanswered questions—about campaigns, designated screening locations, and whether the GP is the correct point of contact—construct a narrative of uncertainty. In this narrative of uncertainty, the healthcare system appears opaque and unresponsive to participants. This questioning tone conveys not only confusion but also an implicit expectation that health services should take a more active, directive role in informing women, suggesting that communication failures are experienced as systemic rather than personal.

By framing the issue collectively (“ladies, you can go…”), she appears to situate herself within a community that she perceives as equally uninformed, subtly critiquing public health outreach that fails to reach women like her. Ultimately, her meaning-making positions breast cancer screening as something dependent on clear institutional guidance. Without such guidance, she appears to experience limited agency, highlighting how structural communication gaps can impede engagement even among individuals who may otherwise be willing to participate. Ultimately, her account shows that breast screening uptake is not only about individual attitudes, but about whether the health system succeeds in reaching, informing, and enabling women from diverse backgrounds. On a slightly similar note, another participant said:

Okay, so for me, I have not gotten any and I would say it’s because there is lack of awareness and in my family, there has not been any case of breast cancer. So, I could say it’s due to lack of awareness (Obiageli).

The preceding extract highlights how the participant frames her non-engagement with breast cancer screening primarily through the lens of a lack of awareness, positioning insufficient knowledge as both the starting point and outcome of her experience. Her repeated emphasis on “lack of awareness” suggests a perceived information vacuum, leaving her without the cues or prompts needed to prioritize screening. By highlighting the absence of breast cancer in her family, she implicitly reveals how personal or familial illness often functions as a catalyst for health-seeking behaviors such, without such a trigger, she relies on external information systems she felt have not reached her. This intertwining of structural and personal factors constructs a lived experience in which breast screening does not emerge as urgent or meaningful, because it has not been rendered salient within her social or informational environment. Her account reflects a broader sense of passivity shaped by the absence of targeted, accessible health communication, underscoring how awareness itself becomes a prerequisite for agency in navigating preventive healthcare.

### Fear of the unknown

This theme reveals how fear and limited information powerfully shape black women’s decisions about breast cancer screening, often leading to prolonged avoidance. It also highlights how cultural context and unfamiliarity with screening practices—intensify uncertainty and emotional resistance. Talking about this, one participant said:

I have not done any screening before. Okay, I can just say maybe that is due to fear or of whatever the results outcome will be. All right, so it’s just due to fear. So, I have not done any screening before (Ifeyinwa).

The participant’s account centers on fear as the dominant force shaping her non-participation in breast cancer screening. Thus, revealing a powerful emotional barrier that appears to override rational awareness of the importance of preventive healthcare. Her repeated assertion that she has “not done any screening before,” coupled with the tentative phrasing “maybe that is due to fear,*”* appears to suggest an internal struggle to articulate or legitimize her anxiety. This hesitancy conveys a sense of vulnerability, as she negotiates the discomfort of acknowledging fear as a determinant of her behavior. The specificity of her concern*—*“whatever the results outcome will be”—illuminates a deeper existential dread, where screening becomes symbolically tied to the possibility of receiving life-altering news. In this way, screening is not simply a routine health action, but an emotionally charged encounter with a potential threat. Her emphasis on fear *“as the”* reason positions this emotion as absolute, revealing a lived experience in which anticipatory distress restricts agency and inhibits engagement. Ultimately, her narrative illustrates how emotional meaning-making can become a significant obstacle. Thus, demonstrating that barriers to screening are not solely structural or informational but can be profoundly internal, rooted in complex feelings about illness, uncertainty, and the implications of knowing. Commenting on fear, another participant also said:

So, for me, I have not done one and I really think […] is due to fear. So, and fear and lack of information really. So back home in Nigeria, I am not sure. There wasn’t quite a lot of information on you going for breast cancer screening, but when I got here. and I got a letter to go for a cervical cancer screening. I delayed it for like a year. I was like, I am not going, I do not even want to know, you know. So, but I ended up after speaking to a medical professional. She was like, oh, there’s no big deal there, just go. So, so I think, yeah, it’s just the general fear, just once you hear cancer, you. (Omolola).

The participant’s narrative reveals a complex interplay between *fear*, *lack of information*, and *cultural transition*, all of which shape her relationship with cancer screening. She repeatedly attributes her non-engagement to fear, suggesting an emotional landscape where screening is associated with threatening knowledge and existential uncertainty*—*“I don’t even want to know.” This avoidance is not presented as irrational but as a deeply felt protective mechanism, intensified by prior experiences of limited cancer awareness “back home in Nigeria,” where screening was not widely publicized or normalized. Her account highlights how migrating into a new healthcare context initially heightened this fear, as seen in her year-long delay of cervical screening even after receiving an NHS invitation. Yet, the turning point in her meaning-making comes through interpersonal reassurance from a medical professional, whose casual normalization*—*“there’s no big deal”*—*helped her reframe screening as manageable. This contrast between informational scarcity in her country of origin and supportive guidance in the United Kingdom underscores how cultural and systemic environments shape emotional responses to preventive care. Ultimately, her meaning-making illustrates how fear is both individually lived and structurally conditioned, with clearer information and relational support emerging as key to overcoming internal barriers.

### From fear to action: empowerment-focused breast screening communication

A notable pattern identified in the participants’ lived experiences was around how fear, misinformation, and cultural context shape black women’s emotional responses to cancer screening, often leading to hesitation or delay. Participants consistently call for communication that feels creative, relatable, and emotionally supportive—using storytelling, survivor narratives, and culturally resonant media to challenge myths and make screening feel less intimidating. At the same time, they emphasize the importance of practical, immediate calls to action, with clear booking avenues, that empower women to move from awareness to confident, timely screening. Commenting on the former, one participant said:

I think they should address common fears and myths, like the idea that screening is only needed when there are symptoms or that cancer always means death. Like if they can use artwork like illustrations, spoken word or short videos, it can help to make the message more emotional and memorable. And I also think it’s important to include encouraging messages from healthcare professionals or community figures. So, people feel reassured and supported. And also, the tone should be positive and empowering, showing that early detection can save lives. Then for me, when it comes to the conclusion, I think the campaign should end with a very strong and clear call to action, like something like encouraging women to book their screening, check their eligibility, or speak to a health care provider, it should also provide simple information on how to take the next step. So, for me, overall, I feel the ending should leave women feeling motivated, informed, and confident enough to take action rather than just scared or overwhelmed. (Otokini).

The participant’s reflections reveal a meaning-making process that appears shaped by a desire for emotionally intelligent, culturally attuned, and empowering health communication, positioning clear information and supportive messaging as antidotes to entrenched fears surrounding breast cancer screening. Her emphasis on addressing “common fears and myths,” and correcting misconceptions that screening is only necessary when symptoms appear, suggests an awareness of how misinformation and collective narratives shape women’s emotional responses to cancer. By advocating for creative, emotionally resonant formats—artwork, illustrations, spoken word, or short videos—she highlights the importance of culturally accessible communication that reaches diverse audiences, particularly those who may not respond to traditional health messaging. Her call for “encouraging messages from healthcare professionals or community figures” underscores the need for relational reassurance, suggesting that trust and emotional safety appear central to enabling women to act.

The participant’s insistence on a “positive and empowering” tone reflects her own lived tension between fear and agency, implying that women are more likely to participate when screening is framed as hopeful rather than threatening. Finally, her perspective for a strong call to action—paired with simple, practical guidance—demonstrates her belief that motivation arises not only from being informed but from feeling capable and supported. Through this account, she constructs screening engagement as an emotional journey. This also include that health campaigns are encouraged to actively shape, moving women from overwhelm toward confidence, clarity, and action. Concerning the content, another participant also said:

I also just wanted to add […] It could be like a video where somebody who has had breast cancer is having fun, so much fun and somebody comes up and like, oh, I thought like you are having fun in a way that somebody that has cancer should not be. And maybe somebody comes up like, Oh, I thought you had cancer. How come you are having this kind of fun and all that? Something catching and then goes into seeing the story or showing a video of, oh, I started early. I did this. This is what I did. It’s all gone. And yeah, something in that line. (Brianna).

The participant’s description reveals a deeply imaginative and emotionally attuned vision of how breast cancer awareness messages should be constructed, reflecting her desire to disrupt the dominant cultural narrative that equates cancer with suffering, limitation and fear. Her idea of a joyful survivor being questioned—captures her attempt to challenge the stereotype that a cancer diagnosis necessarily diminishes a person’s life or identity. Through this imagined scenario, she constructs a counter-story in which survival, early detection, and wellbeing are not only possible but celebratory, suggesting that reframing cancer in this way could offer hope rather than fear.

In making sense of the participant’s comment, her emphasis on something *“catching”* and the transition from light-hearted imagery to a survivor’s testimony reflect an understanding of the emotional journey women undergo when confronted with screening decisions. She appears to be advocating for messaging that first disarms fear and then educates through relatable, humanized storytelling. This imaginative sequence illustrates her own meaning-making: that empowering and normalizing early detection can counteract the dread often associated with cancer. Ultimately, her narrative conveys a belief that campaign messages must not only inform but transform emotional expectations, leaving women feeling that a cancer conversation can be life-affirming rather than terrifying. Another participant, while commenting on the best way to ensure an effective campaign, also said:

About the call to action, I feel like if you put in practical, if I am looking at the social media stuff right now and I have seen all the information on breast cancer and you are like, book your early screening, save your life, book your stuff. How can I book? So maybe like right there on that page, there’s a link that the person can visit, there’s a QR code that they can scan, there’s a phone number to NHS that they can call, you know, something that they can do immediately because once we finish scrolling, we have gone on to other things. We may not end up actually booking. (Omolola).

The participant’s account highlights a deeply pragmatic, experience-grounded understanding of how digital health communication functions within everyday life, revealing her belief that effective screening engagement depends not only on emotional resonance but on immediate, accessible pathways to action. By situating herself within the familiar rhythm of *“scrolling”* through social media, she articulates a lived reality in which attention is fleeting, and intention easily dissipates, making practical facilitation essential. Her rhetorical question*—*“How can I book?”—exposes a gap between motivational messaging and actionable guidance, suggesting that without direct prompts such as clickable links, QR codes, or phone numbers, even well-intentioned individuals may fail to follow through. This insight demonstrates her meaning-making of screening as an activity that requires not only awareness but frictionless navigation, where barriers as small as an absent hyperlink become disproportionately significant in a fast-paced digital environment. Her emphasis on immediacy reveals a recognition that behavior change hinges on capturing momentum before fear, distraction, or daily life intervenes. Ultimately, her reflection positions digital design as an active agent in enabling or obstructing women’s health engagement, emphasizing the importance of campaigns that meet people where they are, simplify decision-making, and transform momentary awareness into concrete action.

### Animated content

A significant superordinate theme emerges around the power of animation to make breast cancer education feel safer, lighter, and more emotionally accessible. Participants describe how art-based animated videos and cartoon characters can reduce fear, challenge the perception of cancer as a death sentence, and reinforce the message that early screening saves lives. Collectively highlighting a strong preference for communication that is both reassuring and empowering, using creative visuals to deliver accurate information while motivating women to engage confidently with screening. Talking about this, one participant said:

I would prefer an animated video. I think an animated video will pass the message across very well. (Otokini).

The participant’s preference for an animated video reflects her belief that accessible, visually engaging formats are central to how health messages are understood and emotionally received, revealing a lived experience in which traditional communication may feel distant or less impactful. Her simple but decisive phrasing—“I would prefer an animated video”*—*suggests an intuitive recognition that animation can soften the emotional weight often associated with breast cancer communication, making the message feel safer, less clinical, and more approachable. This preference indicates that she views animated content as capable of breaking down complex or frightening topics in a way that feels digestible and non-threatening, aligning with her broader concerns about fear, reassurance, and emotional readiness.

In expressing that animation would “pass the message across very well,” she appears to be articulating a desire for communication that resonates across cultural contexts and literacy levels, suggesting that visual storytelling can bridge gaps that written or medicalized information cannot. Her meaning-making positions animation as a tool that not only educates but also creates emotional distance from fear, enabling women to engage with screening messages without feeling overwhelmed. Ultimately, her account highlights the importance of the medium as part of the message, showing how the format of communication shapes whether women feel able to absorb and act on information about breast cancer screening. Commenting on animation, another participant also said:

Animated video will be better because with art-based animated videos you can make light an otherwise complex topic related to breast cancer. Using like cartoon character can also help reduce the fear associated with breast cancer while also passing the message across that early screening save life and breast cancer is not a death sentence (Teyana).

The participant’s preference for an animated, art-based video reflects a deeper meaning-making process in which she seeks to transform the emotional landscape surrounding breast cancer from one dominated by fear into one that feels approachable, hopeful, and humanized. By emphasizing that animation can “make light an otherwise complex topic,” she reveals an understanding that breast cancer communication often feels heavy, intimidating, or overwhelming, and that softening the tone can create emotional safety for women who may already feel apprehensive about screening. Her suggestion to use cartoon characters suggests that visual storytelling can reduce the fear associated with cancer, enabling viewers to engage with the message without the psychological burden that clinical or realistic imagery may evoke.

This reflects her lived experience of fear as a significant barrier and her desire for messaging that counters the cultural association of cancer with fatalism. Her statement that animation can still effectively convey that “early screening saves lives” and that “breast cancer is not a death sentence” highlights her conviction that empowerment and reassurance must be central to awareness campaigns. Ultimately, the participant constructs an animated video not only as a communication tool but also as an emotional intervention—one capable of reframing cancer from a terrifying possibility into a survivable, manageable reality through gentler, imaginative, and psychologically accessible storytelling.

### Length

This theme centered on the need for breast cancer communication to align with the fast, fragmented rhythms of digital attention. In this theme, participants frame short, visually engaging videos as essential for sustaining interest, while drawing on their own scrolling habits to illustrate how quickly longer content becomes disengaging. The emphasis on brevity, catchiness, and supportive captions reflects a deeper belief that effective health messaging is encouraged to conform to the realities of modern media use, offering information in formats that are immediate, accessible, and capable of holding attention in crowded online spaces. Talking about this, one participant said:

For me, I would say 2 min. It has to be very catchy for, well, I am speaking from the kind of person that I am. If it is like 3, 4 min, it’s getting too long for me to stay engaged in it for that long. So, 2 min or 1.5 min, if they can put all the information and probably a caption that has the link to book breast cancer screening (Brianna).

The participant’s emphasis on a short, *“catchy*” video reflects a clear awareness of how limited attention spans and fast-paced digital habits shape engagement with breast cancer messages. By grounding her preference in her own experience, she highlights the need for brief, visually appealing content that can compete with the rapid flow of social media. In making sense of her lived experience, her call for concise captions and layered communication underscores the belief that effective screening messages must be immediately accessible, emotionally engaging, and easy to absorb within the distractions of online life. Another participant commenting on the importance of brevity also said:

What I am really bent on is just, it should be short and precise because people have like very short retention span when it comes to like social media. They just want to see something very short and move on. You understand what I mean? And when it is becoming long, it becomes like more boring. People do not have that time. (Oroti).

The participant’s insistence that health messages be “short and precise” reflects her lived understanding of how attention functions within fast-paced social media spaces, where content is consumed quickly and easily abandoned. By grounding her view in her own habits, she constructs a shared narrative of limited attention spans, suggesting that people “just want to see something very short and move on” because of constant informational overload. Her belief that longer videos become “*boring*” and that “people don’t have that time” positions brevity as a psychological necessity rather than a stylistic choice, highlighting how fragile engagement can be online. Ultimately, she frames concise, visually engaging communication as a form of respect for the audience’s reality, arguing that effective breast cancer messaging must align with contemporary patterns of digital attention to genuinely influence behavior.

### Embedding awareness in women’s everyday digital spaces

A notable theme in the participants’ narrative emerges around the strategic use of highly visual, high-traffic digital platforms to maximize the reach and impact of breast cancer messaging. The participant consistently emphasizes placing short, engaging videos within the everyday media environments women already inhabit—whether on TikTok and Facebook, or on platforms like YouTube and Netflix, where brief ads appear during entertainment and are harder to skip. Together, these accounts frame effective communication as content that meets women directly within their habitual digital spaces, using concise, visually compelling formats to ensure the message is both seen and absorbed. Commenting on this, one participant said:

I would suggest using platforms like Instagram, Facebook and TikTok to share the campaign. Although for me personally, I feel Instagram is really more effective because it’s very visual, you know, and so it works well for ad-based content like images, short videos and graphics. And a lot of women spend time there and it’s easy to share posts, stories and reels that can reach a wide audience. (Otokini).

As the preceding extract shows, the participant’s preference for platforms like Instagram, Facebook, and TikTok reflects her understanding of where women in her community naturally spend their time and how they engage with visual information. By emphasizing Instagram as “really more effective” because it is highly visual and supports images, graphics, and short videos, she reveals a meaning-making process that links visual media with emotional resonance and ease of understanding. In making sense of her experience, her focus on shareability—stories, posts, and reels that can “reach a wide audience”—appears to suggest that she views breast-cancer awareness as spreading best through everyday digital interactions, not through formal health channels. Her narrative suggests that effective communication appears required to meet women where they already are online, using familiar, visually engaging formats that feel approachable, culturally relevant, and easy to circulate, making awareness feel woven into the rhythms of everyday social media use. Another participant also said:

So, I would suggest TikTok and Facebook because a large number of women actually spend a good number of hours on TikTok. So, I feel by using TikTok, they’ll be able to message to be able to get across to them using that platform. (Omonigho).

The participant’s suggestion to use TikTok and Facebook reflects her understanding of where women in her community spend significant time and how digital habits shape engagement with health messages. By highlighting that “a large number of women actually spend a good number of hours on TikTok,” she draws on her lived experience of contemporary media use to interpret these platforms as natural entry points for breast-cancer communication. Her reasoning appears to show that effectiveness depends on meeting women within the rhythms of their daily online lives, where familiarity and frequent use increase the likelihood of meaningful contact with health information. This meaning-making positions social media not just as a distribution tool but as a social environment where awareness can spread organically and feel more relevant, accessible, and aligned with women’s everyday routines. Another participant also said:

If we just have like a 30 s video that goes on YouTube into a video or even Netflix does that too, where you are watching and then an ad pops up, but because you are enjoying the movie, you have to wait for the ad, so that might also be another platform. (Omolola).

The participant’s suggestion to use short YouTube or streaming platform adverts reflects her lived understanding of how people encounter information in everyday digital spaces. By imagining a 30 s ad that appears while someone is already engaged in a video, she highlights how attention can be captured most effectively when viewers are already *“locked in”* and waiting for content to resume. This meaning-making positions advertising interruptions not as nuisances but as strategic moments when individuals are more likely to watch a message in full. Her account suggests that for breast cancer awareness to cut through distraction, campaigns must integrate into the natural media habits people already have, using brief, unavoidable touchpoints to deliver essential information.

### Empowerment through coproduction, inclusive voice and agency

The preceding theme centered on empowerment, ownership and inclusion within the coproduction process. Participants describe feeling genuinely part of the solution to the persistent inequalities in early breast cancer screening for Black women, highlighting how the process gives them a meaningful voice in shaping interventions that directly affect their lives. Their reflections emphasize the emotional significance of being heard, valued and represented in a space where their perspectives have historically been overlooked. Together, these accounts position coproduction not just as a method, but as an affirming experience that restores agency and visibility to Black women in health research. Talking about this, one participant said:

I think is very helpful. Very helpful because, okay, all this information is needed to be able to build the right content to reach out to people. And we need to just get it right to get them to be able to reach out to the right audience, if not, one might just do and get a content that will not really be useful. So, I think this is very good. (Amenze).

The participant’s reflections reveal a strong sense of appreciation for the process, interpreting the gathering of information as essential for creating content that genuinely reaches the right audience. Her emphasis on getting it *“right”* suggests an underlying concern that poorly designed health messages can easily miss their target, rendering them ineffective. This meaning-making shows an awareness of the complexity involved in crafting breast-cancer awareness campaigns and a belief that thoughtful consultation prevents wasted effort. Her repeated affirmations that the process is *“very helpful*” and *“very good”* indicate that being included in shaping the message feels purposeful and necessary, reinforcing her sense that meaningful health communication must be informed by the lived experiences of the people it aims to serve. On a similar vein, another participant said:

Just an appreciation because thank you so much for doing this. It’s been very intentional. We know that most of the criteria for severe interventions or research that has been done in the United Kingdom especially those on breast cancer do not really cover most people from other minority groups like the black community. And to see something that is so focused on how we perceive it, and how we want it to be done sounds really intentional and thank you for taking up the responsibility to do it. (Owasémé).

As the preceding extract shows, the participant’s expression of appreciation reveals a deep sense of meaningful inclusion in a process she feels has historically overlooked people from minority backgrounds. By emphasizing the *“intentional”* nature of the work and contrasting it with United Kingdom breast-cancer research and interventions that “do not really cover” minority groups, she constructs this experience as unusually validating and responsive to her community’s needs. Her repeated gratitude reflects a lived experience of marginalization in health research, where people like her often feel unseen or excluded, underscoring the significance and personal meaning of this initiative. The phrase “focused on how we perceive it, and how we want it to be done” seems to suggest she interprets the project as one that centers Black women’s voices, perspectives, and cultural contexts, fostering a sense of empowerment and recognition. Ultimately, her account suggests that being asked to contribute is not merely participatory but restorative, allowing her to feel represented in a space where representation has long been lacking. Another participant also said:

Overall, being part of this coproduction process makes me feel part of the solution to poor early breast cancer screening for black women. It gives me a sense of ownership to contribute to the solution, especially given my lived experience of never having gone for breast cancer screening. I am truly happy to be part of this inclusive process that takes a bottom-up approach and gives a voice to black women like me, who are often not heard. (Teyana).

The participant presents their involvement in the coproduction process as deeply empowering, interpreting it as a rare opportunity to contribute to addressing the low rates of early breast cancer screening among Black women. By stating that the process makes her feel “part of the solution,” the participant expresses a shift from passive exclusion to active ownership, grounded in her lived experience of never having attended screening herself. This sense of ownership is tied to a broader narrative of historical underrepresentation, as they appear to highlight how research and interventions in the United Kingdom often overlook minority groups. The participant’s appreciation reflects a profound recognition of being intentionally included in a bottom-up, community-centered approach that values voices like hers, which are often not heard. Through this meaning-making, the participant experiences the process as informative and also socially and personally validating, reinforcing a sense of collective responsibility and belonging in shaping more inclusive health solutions.

## Discussion

The purpose of the study was to examine how black women who are partially or not up to date with breast cancer screening could inform the development and co-production of an art-based media intervention that effectively promotes early screening uptake in the United Kingdom. Based on the analyzed dataset, several themes emerged which will be discussed in relation to the literature.

Firstly, the findings of this study reveal how Black women’s engagement with breast cancer screening in the United Kingdom appears shaped by an interplay of emotional, informational, cultural, and structural factors, echoing patterns consistently emphasized in the literature. Participants described *fear*—particularly fear of diagnosis and its imagined consequences—as one of the primary barriers to screening, aligning with evidence that emotional responses, including fear, stigma, and anxiety, are key inhibitors for Black African and Black Caribbean women (8). Fear was often intertwined with *limited awareness* and uncertainty about how the screening system works, reflecting wider gaps in knowledge and awareness reported among minority ethnic women in the United Kingdom (22). Consistent with research showing that many Black women possess “low perceived risk” and uncertainty around screening pathways (23), participants in this study described not knowing where to go, how to book, or even what screening involves, with some linking this to prior experiences of minimal cancer education in their countries of origin. Our finding contrasts with the finding from previous studies, which show that white women are more likely to engage in breast cancer screening compared to black women in the United Kingdom (24). Our findings therefore underscore the key barriers behind the disparities in black women’s poor engagement in screening and the need to address fear to encourage early screening uptake.

Our study also found that breast cancer awareness campaigns often fail to reach or represent Black communities, closely mirroring national evidence showing that existing research and interventions rarely center Black women’s perspectives or address their specific emotional and cultural barriers (12). This historical exclusion surfaced in expressions of gratitude for being included in a coproduction process that *“feels intentional,”* revealing how meaningful it is for participants to see their cultural experiences recognized. Such sentiments align with calls in the literature for more culturally sensitive, community-driven interventions that acknowledge diverse beliefs, stigma, and communication needs (8, 22).

Secondly, the findings highlight that women’s preferred communication styles are shaped by their lived experiences of digital media. Participants consistently advocated for short, visually appealing, and emotionally safe content—especially animation, relatable storytelling, and positive survivor narratives—as strategies to counter fear and enhance retention. This aligns with research emphasizing that effective interventions must address emotional barriers, misconceptions, and cultural narratives that equate cancer with death or hopelessness (8). The preference for platforms such as Instagram, TikTok, and YouTube demonstrates how social media habits shape health engagement and how digital environments can serve as meaningful sites for culturally tailored cancer communication. Participants’ insistence on accessible “calls to action”—including links, QR codes, and direct booking prompts—further emphasizes the need for practical, low-friction pathways to screening, consistent with behavior-change models that highlight capability, motivation and opportunity as key determinants of screening uptake (25). Regarding the need for brevity in media messages, our findings resonate with a previous study drawing on data from 11 Nigerian women not up to date with breast cancer screening who emphasized the need for concise, compassionate messaging to motivate early screening (26). Regarding awareness, our study findings are consistent with Bulut and Gok (27), which examines the roles of supportive systems, self-efficacy, and motivation in breast cancer and draws on cross-sectional data from 308 women. Bulut and Gok (27) conclude in their study that there is a need to strengthen awareness, including self-efficacy, to improve early detection and preventive behaviors. The need for the media’s role in creating awareness and fostering action was further emphasized in Bulut and Bardak’s (28) study in shaping women’s self-examination behavior.

Thirdly, the findings highlight how emotional fear, cultural stigma, and practical barriers intersect to undermine early breast-cancer screening uptake among Black women in England. Participants’ emphasis on transforming fear-based messaging into empowering, human-centered communication reflects wider evidence that Black women often experience heightened anxiety about cancer, shaped by fatalistic beliefs and community narratives that equate diagnosis with death (11). Our findings also reveal the impact of stigma, particularly the perception that cancer survivors should appear unwell, which aligns with research showing that cultural expectations and misconceptions within some Black communities contribute to delayed help-seeking (29). Furthermore, the call for immediate, practical booking options echoes studies demonstrating that structural barriers—such as digital exclusion, lack of clear information, and competing life pressures—disproportionately affect Black women and reduce follow-through after initial engagement (NHS (30)). Together, these insights reinforce the need for screening campaigns that are emotionally resonant, culturally sensitive, and behaviorally realistic, offering both reassurance and simple, immediate pathways to action.

Finally, our study found that participants articulated a profound sense of empowerment through the coproduction process, describing it as inclusive, validating, and identity-affirming. Their reflections resonate with growing evidence that participatory, community-embedded approaches are essential for improving screening outcomes among underserved groups, who often face cultural stigma, communication barriers, and negative experiences within health services (31). Feeling “part of the solution” suggests that the process itself may serve as an intervention, strengthening agency, awareness, and trust. This aligns with the principles of co-production and the involvement of those directly affected to ensure their voices are heard and to directly inform the interventions needed to address the challenges they face (32).

Collectively, the findings reinforce the need for breast cancer screening interventions that are emotionally attuned, culturally grounded, co-designed with communities, and delivered through familiar digital channels. They also highlight a critical gap echoed across the evidence base: while Black women in the United Kingdom face disproportionate burdens of late diagnosis and low screening uptake, the interventions designed to address these inequalities often fail to adequately target their specific needs. This study contributes to addressing that gap by centering Black women lived experiences, demonstrating how coproduction can illuminate barriers invisible in conventional top-down approaches and forge pathways toward more equitable early detection.

### Strengths and limitations

A key strength and original contribution of this study is its use of Interpretative Phenomenological Analysis (IPA) within a coproduction framework, enabling rich, in-depth insights into how Black women make sense of poor breast cancer screening uptake. Coproduction approaches remain limited within United Kingdom breast cancer research, despite evidence that Black women’s perspectives are routinely underrepresented in screening studies and intervention design (33). By centering the lived experiences of women who are *eligible* but have *not* engaged in screening—a group largely absent from previous research (8)—the study addresses a recognized gap and contributes a new understanding of emotional, cultural, and informational barriers. The diversity of experiential reflections, including fear, cultural stigma, media habits, and preferences for communication formats, provides nuanced insight that informs media campaigns to encourage uptake. Another strength lies in the depth of engagement: participants expressed feeling seen, represented, and empowered through the process, suggesting the methodology itself fostered trust, authenticity, and richer disclosure—key markers of high-quality IPA work.

However, the study also has limitations. As with all IPA research, the small sample size restricts generalizability; the aim is depth rather than breadth. However, wider patterns across other ethnic minority communities, such as South Asians, cannot be assumed. In essence, while the study surfaced strong emotional and cultural meanings around screening, it did not formally compare experiences across other ethnic groups, such as South Asian women who may face distinct types of stigma, fear, and health-system barriers. Another limitation is that given the qualitative nature of the study, it did not measure confounding variables that might have impacted participants’ views, such as regional economic shifts or specific participants’ biases that might influence the findings. Overall, the study’s strengths lie in its depth, inclusivity, and methodological alignment with the communities it seeks to support. These insights offer a strong foundation for developing culturally responsive, emotionally attuned interventions for improving early breast-cancer screening among Black women in the United Kingdom.

### Policy implications

Our study has important policy implications for policymakers, in that it highlights the need for United Kingdom breast-cancer screening policy to move beyond generic, top-down communication and adopt culturally responsive, community-driven strategies that address the emotional, informational, and structural barriers faced by Black women. Participants’ experiences of fear, stigma, and limited awareness directly reflect national evidence showing that Black African and Black Caribbean women encounter distinct psychosocial barriers, misinformation, and gaps in cancer knowledge that current interventions fail to address (8, 22). Policy is therefore encouraged to prioritize the integration of co-production into screening design, ensuring that Black women—particularly those who are eligible but do not attend—are meaningfully involved in creating campaigns that reflect their lived experiences, a gap repeatedly identified in their perceived non-involvement as shown in our study.

In line with our findings, policymakers are encouraged to fund short, visually engaging digital content—such as animation, survivor storytelling, and myth-busting messages—optimized for platforms like Instagram, TikTok, Facebook, YouTube, and streaming adverts, where participants report Black women spend substantial time. These campaigns are required to incorporate immediate calls to action—QR codes, direct booking links, and NHS helpline numbers—to reduce friction in booking and counter the fleeting nature of social-media attention. Collectively, these recommendations call for a policy shift that centers cultural nuance, emotional safety, and community partnership as core components of equitable breast-cancer early-detection strategies.

## Conclusion

Breast cancer survival rates disproportionately affects black women in the United Kingdom, who face a high risk of mortality due to late presentation and poor early screening uptake. In conclusion, this study advances understanding and demonstrates that improving early breast cancer screening uptake among Black women in the United Kingdom requires confronting an interconnected set of emotional, cultural, and structural barriers that have been repeatedly overlooked in mainstream research and policy. Through IPA and coproduction, participants revealed how fear, low awareness, stigma, and unfamiliarity with screening pathways mirror national findings showing that Black African and Black Caribbean women face distinct psychosocial and informational obstacles to participation (8, 22). At the same time, their strong preference for culturally resonant, visually engaging digital communication underscores the need for more tailored, community-centered approaches that meet women where they are—aligning with calls for culturally sensitive interventions that speak to lived experience. Crucially, the sense of empowerment and representation expressed by participants highlights the transformative potential of coproduction to address disparities that stem, in part, from historical exclusion in health research and service design. Together, these insights reinforce that meaningful progress in screening equity will depend on embedding cultural nuance, emotional attunement, and community voice at the heart of future interventions, policies, and health communication strategies.

## Data Availability

The original contributions presented in the study are included in the article/supplementary material, further inquiries can be directed to the corresponding author.
